# Prevalence and network analysis of depressive, anxiety and cognitive symptoms among oldest-old population in China: A cross-sectional study

**DOI:** 10.1097/MD.0000000000050233

**Published:** 2026-08-14

**Authors:** Quan Zhou, Qianyu Zhao, Boning Zhang

**Affiliations:** a School of Life and Health Sciences, Environmental Engineering, Hefei University, Heifei, Anhui Province, China; b Graduate School of Translation and Interpretation, Beijing Foreign Studies University, Beijing, China; c School of English Studies, Beijing International Studies University, Beijing, China.

**Keywords:** anxiety, cognitive impairment, depression, network analysis, oldest-old population

## Abstract

The oldest-old people are prone to such psychological and mental risks as depression, anxiety, and cognitive dysfunction. Yet which specific symptom poses the biggest threat remains elusive, especially when the characteristics of a certain sample are considered. Using network analysis, the current study examined a pool of symptoms in the Chinese Longitudinal Healthy Longevity Survey and established a network model. Depression was more prevalent (67.4%) among the oldest-old Chinese than anxiety (11.2%). Depressive, anxiety, and cognitive symptoms exhibited very weak interrelations, and “feeling depressed” was the most prominent symptom in the whole network model. Based on such findings, interventions should be targeted at depressive symptoms to improve mental health for this population.

## 1. Introduction

Over the past decades, China has experienced a rapid process of population aging. In a broad sense, the old population could refer to anyone aged 60 and above. Academically, however, the old population should be further divided into 3 subgroups, that is, young-old adults (age 60–70 years), old-old adults (age 70–80 years), and oldest-old adults (age 80 years and over), as studies have shown that there are significant sociodemographic differences between these subgroups.^[1,2]^ According to projections from the United Nations, the oldest-old group accounted for 18.0% of the global old population.^[3]^ In China, the oldest-old represent a highly vulnerable group. They are among the most policy-neglected and socially disadvantaged populations, facing significant challenges in health, social support, and access to resources. A large body of research has indicated that the oldest-old population has unique risk factors and clinical presentations compared with other subgroups of old citizens,^[4]^ yet they remain underrepresented in the literature. Moreover, there is increased heterogeneity in the group of people aged 80 and above, making symptoms more complicated for this particular group.^[5]^ Such changes and characteristics necessitate a comprehensive analysis of the public health risk faced by the oldest-old Chinese citizens.

The oldest-old group is often beset by such psychological problems as anxiety and depression, which normally appear as complications of other diseases. For example, around 25% of myocardial infarction victims are diagnosed with major depression, and another 25% with minor depression.^[6]^ Apart from psychological problems, the oldest-old population is also prone to various mental illnesses and diseases. Common illnesses include delirium, dementia, and psychosis, among others.^[7]^ Another mental health risk related to aging is cognitive impairment, which compromises old people’s literate, numerical, attentional, mnemonic and other capabilities, putting huge burdens on their daily life. Specifically, elders with moderate-to-severe cognitive impairment are 27% more likely to die in the following 15 years.^[8]^ China-specific evidence further underscores the need to examine these domains together in advanced old age. Nationally representative studies have reported a substantial burden of depressive symptoms among Chinese older adults,^[9,10]^ while aging research indicates that cognitive impairment becomes increasingly common with advancing age.^[11]^ In the Chinese context, widowhood, living arrangements, rural–urban resource differences, and uneven access to social and medical support may further shape late-life emotional and cognitive health. These factors are especially salient for oldest-old adults, who often experience compounded physical frailty, bereavement, and dependency compared with younger-old adults.

Expectedly, psychological, mental, and cognitive problems should influence senior citizens in a complicated interactive way, and these problems or illnesses themselves would affect each other in some way too. In reality, however, evidence in this respect shows diverging results. For example, a community-based longitudinal study covering 3107 elderly Dutch citizens showed that there was a curvilinear relationship between anxiety symptoms and cognitive performance (i.e., mild anxiety could benefit cognitive functioning while severe anxiety only harms cognition), whereas depression symptoms all had a negative impact on old people’s cognition.^[12]^ Specifically, depression adversely influences cognition in terms of processing speed, verbal fluency, and episodic memory, while anxiety mainly affects verbal fluency.^[13]^ Yet some researchers contend that such influence of depression on cognition is unidirectional,^[14]^ or even not tenable at all.^[15]^ Therefore, the complex interrelations between depression, anxiety, and cognition among oldest-old people are far from clear and need to be addressed. The current paper aims to help disentangle the many different symptoms of depression, anxiety, and cognition among oldest-old Chinese citizens using network analysis.

Network analysis is an innovative methodology that has gained widespread application in visualizing structural relationships across diverse domains. Over recent decades, it has been employed in fields such as sociology, economics, and, most notably, psychology.^[16]^ In psychological research, network models conceptualize nodes as individual components – such as symptoms or psychological traits – and edges as the associations between them, with connection strength quantified through weighted parameters derived from empirical data. This framework enables a nuanced examination of the complex interrelations among variables linked to specific outcomes. Moreover, it facilitates the identification of central nodes and key connections, which may serve as strategic targets for intervention and the improvement of health-related outcomes.^[17]^ Importantly, network theory posits that observed associations among symptoms may reflect direct interactions rather than merely manifestations of an underlying latent disorder.

Previously, network analysis has been applied to assess interrelationships among psychosocial and cognitive symptoms in older adults. For example, studies have found that older adults showed weaker connectivity between depression and anxiety symptoms than younger adults,^[18]^ that sad mood was the strongest factor that bridged depression and cognition modules,^[19]^ and that depressive symptoms and cognitive deficits mutually predicted each other.^[20]^ However, these studies did not simultaneously integrate depressive, anxiety, and cognitive symptoms in a symptom-level network focused on Chinese adults aged 80 years and above. The oldest-old group differs from younger-old adults in frailty, comorbidity, bereavement, functional dependency, and survival selection, which may alter symptom prevalence and symptom-to-symptom connections. To address this gap, the current paper explored interrelationships between 3 different domains of depressive, anxiety, and cognitive symptoms among oldest-old Chinese adults using network analysis.

## 2. Materials and methods

### 2.1. Participants

This study was a secondary analysis using data from the 2017 to 2018 wave of the Chinese Longitudinal Healthy Longevity Survey, which is a nationally representative survey focusing on the physical and mental health of Chinese older adults (Center for Healthy and Development, 2020). The survey employed a multi-stage, random sampling strategy to recruit participants from approximately two-thirds of China’s provinces. Detailed descriptions of the study design and methodology are available in previous publications.^[21,22]^ Participants included in this analysis met the following criteria: aged 80 years or older; and completed assessments for basic sociodemographic characteristics, depressive symptoms, anxiety symptoms, and cognitive performance. Exclusion criteria were: age younger than 80 years; missing item-level data required for CES-D-10, GAD-7, or MMSE domain scores; and missing key sociodemographic information required for descriptive analysis. No additional exclusions were made on the basis of comorbid physical disease or low MMSE score, because cognitive impairment was one of the principal domains of interest. The study protocol was approved by the Biomedical Ethics Committee of Peking University (IRB00001052-13074), and all participants provided written informed consent.

### 2.2. Measures

Basic sociodemographic characteristics were collected through self-reported questionnaires, including age, gender, marital status, education level, living arrangement, physical activity, and current smoking and drinking status.

Depressive symptoms were assessed using the validated Chinese version of the CES-D-10.^[23-25]^ This scale evaluates the frequency of depressive symptoms experienced over the past week. Each item is rated on a 4-point Likert scale (0 = rarely or none of the time to 3 = most or all of the time), with a total score ranging from 0 to 30. Items 5, 7, and 10 are scored in reverse. The CES-D-10 covers affective symptoms (e.g., depressed mood and hopelessness) and somatic or behavioral symptoms (e.g., sleep disturbance and effort), and previous validation studies in Chinese older adults have supported its reliability (Cronbach’s α around 0.8) and validity (sensitivity 0.92, specificity 0.87).^[24,25]^ Consistent with prior Chinese studies, a score of 10 or above was considered “having depression.”^[24]^ Note that although CES-D-10 covers affective and somatic symptoms, it’s overall a unidimensional measure.

Anxiety symptoms were assessed using the validated Chinese version of the 7-item Generalized Anxiety Disorder scale (GAD-7).^[26-28]^ This instrument evaluates the frequency of anxiety-related symptoms over the past 2 weeks across 7 domains; each item is rated on a 4-point Likert scale ranging from 0 (“not at all”) to 3 (“nearly every day”), yielding a total score between 0 and 21. The GAD-7 focuses on core anxiety features, including nervousness, uncontrollable worry, generalized worry, trouble relaxing, restlessness, irritability, and fear. Previous Chinese validation work has shown good reliability (Cronbach’s α around 0.9) and convergent validity (*r* = 0.72 with HADS-A) for the GAD-7.^[28]^ Higher scores indicate greater severity of anxiety symptoms. A score of 5 or above was considered “having anxiety.”^[27]^

Cognitive function was measured using the validated Chinese version of the Mini-Mental State Examination (MMSE).^[29]^ The MMSE consists of 24 items covering 6 cognitive domains: orientation (10 items), registration (3), attention and calculation (5), recall (3), and language (8). Total scores range from 0 to 30, with higher scores indicating better cognitive performance. The Chinese MMSE has been widely used in Chinese elderly populations and shows acceptable reliability (Cronbach’s α around 0.88) and validity for screening cognitive impairment (sensitivity 0.87).^[29,30]^ Participants with scores below 25 were considered to have mild cognitive impairment.^[30]^

### 2.3. Statistical analysis

The network structure of depressive, anxiety, and cognitive symptoms was constructed using the R program. The network model was built using the Graphical Gaussian Model; the edges within the network model were regularized via the graphical least absolute shrinkage and selection operator and selected based on the Extended Bayesian Information Criterion.^[31]^ In the network model, each item was depicted as a node, and the edges between nodes represented associations. The thickness of the edge reflected the strength of the association, while the color indicated its direction – green for positive and red for negative relationships. Node centrality within the network was quantified using the Expected Influence (EI), which captures both the positive and negative associations surrounding the node.^[17]^ The index of EI reflects the ability of the specific node in the activation and maintenance of other symptoms within the network model.

The robustness of the network model was evaluated from both EI and edge weights. Correlation stability coefficients (CS-C) for EI were calculated to assess the reliability of centrality metrics, with values above 0.25 (preferably >0.5) indicating acceptable stability.^[32]^ The precision of edge weights was examined via nonparametric bootstrapping to generate 95% confidence intervals (CIs), where narrower intervals signified more reliable estimates. Bootstrapped difference tests were also conducted to determine the statistical significance of differences in node EI and edge weights.

All analyses were performed using the R program (R Core Team, 2021). *NetworkTools* and *qgraph* packages were used to generate and visualize the network models.^[32,33]^ The stability of the network centrality index was examined by the package *bootnet*.^[33]^

## 3. Results

### 3.1. Participant characteristics

A total of 15,874 participants participated in the survey and provided consent, and finally, 4,747 oldest-old participants met the inclusion criteria. The average age of participants was 90.27 (standard deviation = 7.28) years.

Table 1 shows the demographic characteristics of participants. Around half of the participants were male (N = 2,080, 43.8%). While the majority of participants were educated (N = 2,083, 43.9%) and lived with others (N = 3,591, 75.6%), less than half of them were married (N = 1,409, 29.7%). In terms of lifestyle information, only 631 of them were current smokers (60.7%), and 613 were current alcohol drinkers (12.9%). The prevalence of depression (CESD-10 cutoff value of ≥10) was 67.4% (95% confidence interval (CI) = 66.02–68.69%), and the prevalence of anxiety (GAD-7 cutoff value of ≥5) was 11.2% (95% CI:10.34–12.15%), while 36.9% (95% CI: 35.57–38.31%) had mild cognitive impairment.

**Table 1:** Demographic characteristics of participants (N = 4747).

Variables	Total sample	Percentage (%)
Male	2080	43.81
Married	1409	29.7
Registered rural residents	1920	40.4
Living with others	3591	75.6
Educated	2083	43.9
Regular physical exercise	1450	30.5
Current cigarette smoker	631	13.3
Current alcohol drinker	613	12.9
Depression (CESD-10 total ≥ 10)	3198	67.4
Anxiety (GAD-7 total ≥ 10)	101	2.1
Cognitive impairment (MMSE total ≤ 14)	1753	36.9

	Mean	SD
Age (yr)	90.27	7.28
CESD-10 total	12.48	6.04
GAD-7 total	1.35	2.67
MMSE total	24.45	5.95

CESD-10 = 10-item Center for Epidemiologic Studies Short Depression Scale, GAD-7 = 7-item Generalized Anxiety Disorder, MMSE = Mini-Mental State Examination, SD = standard deviation.

### 3.2. Network model

**Figure 1.**
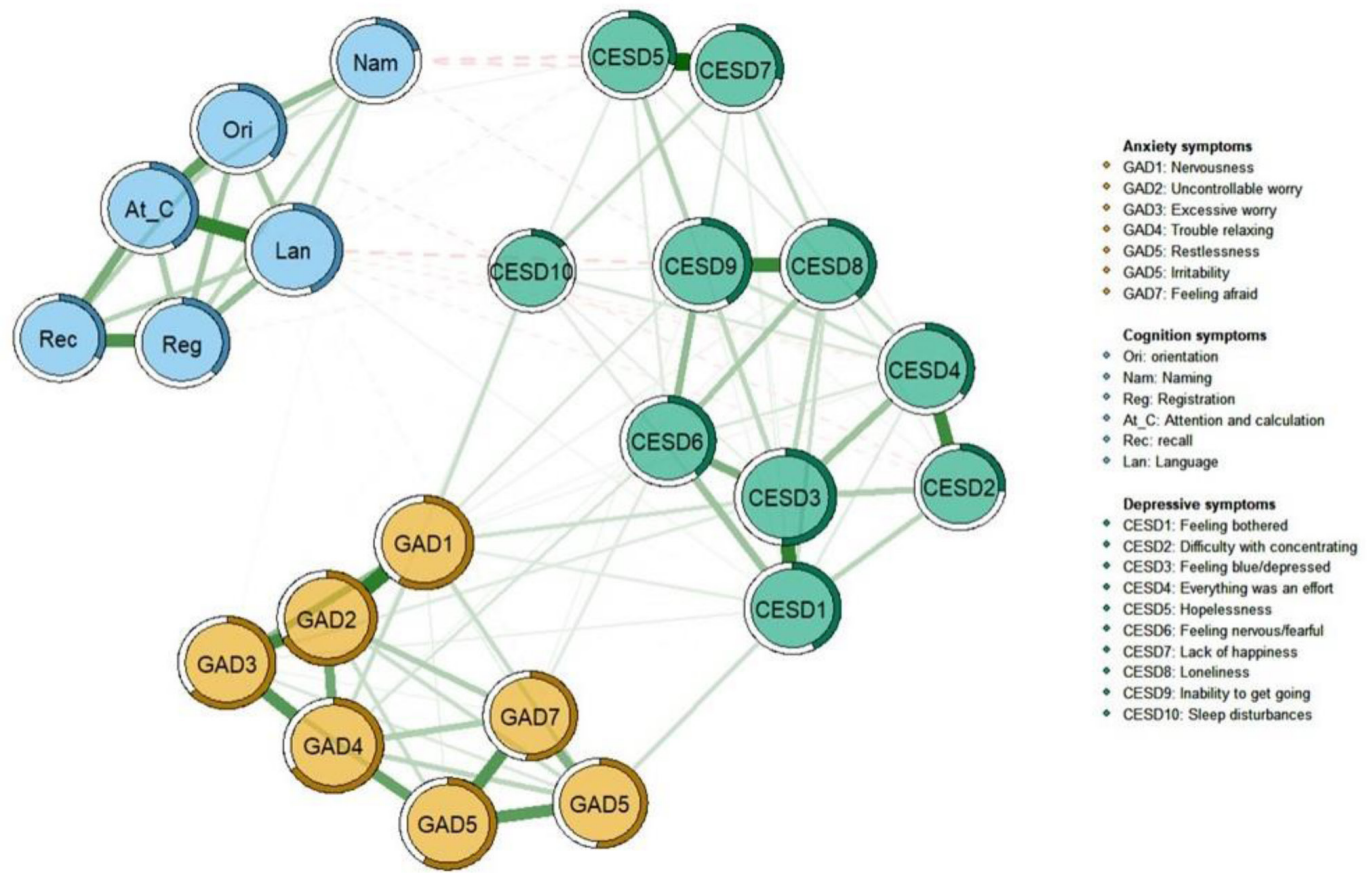
Network structure of depressive and anxiety symptoms and cognition among the oldest-old Chinese population.

Figure 1 depicts the network structure of depressive, anxiety, and cognitive symptoms. Of 253 potential edges, 138 were selected into the network model, with a mean edge weight of 0.036. The edges within the same cluster are marked in green, which indicates that associations of nodes from the same mental disorder were positive. The darker the lines, the stronger the connections. In addition, the associations across different clusters show different patterns. Specifically, the associations between depressive (the green cluster) and anxiety (the yellow cluster) symptoms are positive, while the associations between depressive and cognitive clusters are negative, as indicated by the red dashed lines.

**Figure 2.**
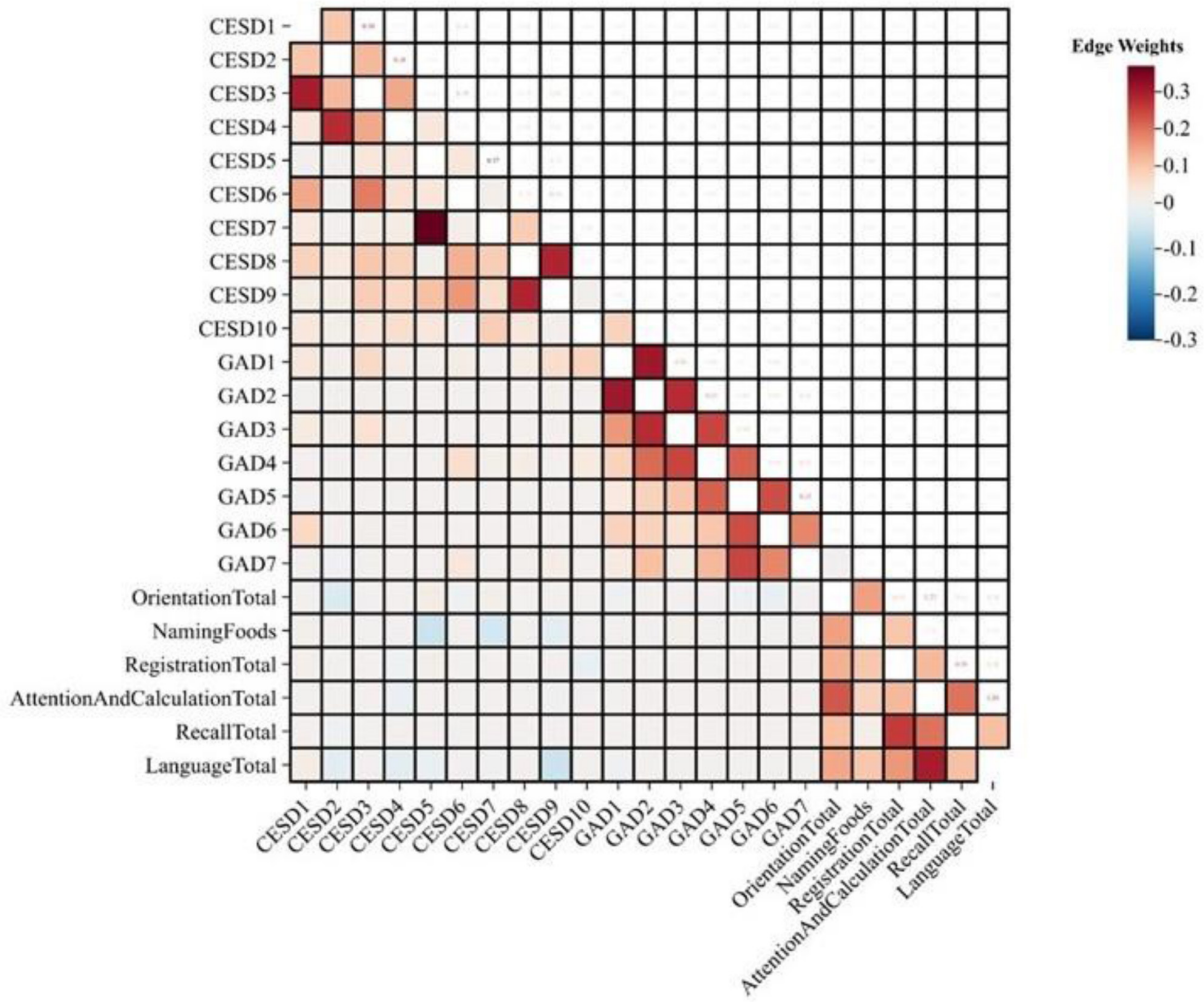
Weighted adjacency matrix of the depressive and anxiety symptoms and cognition network among the oldest-old population.

Figure 2 shows the details of edge direction and strength. Generally speaking, the absolute values of inter-cluster edge weights are all below 0.3, implying very weak connections, if any, among the 3 clusters, regardless of the direction. Moreover, there are obvious negative relations between older people’s cognition and depressive symptoms, as is shown in the light blue cells in the bottom left part. For example, participants’ food naming ability is negatively associated with their hopelessness (CESD5), and their total language ability is also negatively related to their inability to get going (CESD9). The strongest positive connection is found between nervousness (GAD1) and sleep disturbance (CESD10), with an edge weight of 0.08, while the strongest negative bond is found between total language ability and inability to get going (CESD9), with an edge weight of −0.06.

### 3.3. Network centrality and stability

**Figure 3.**
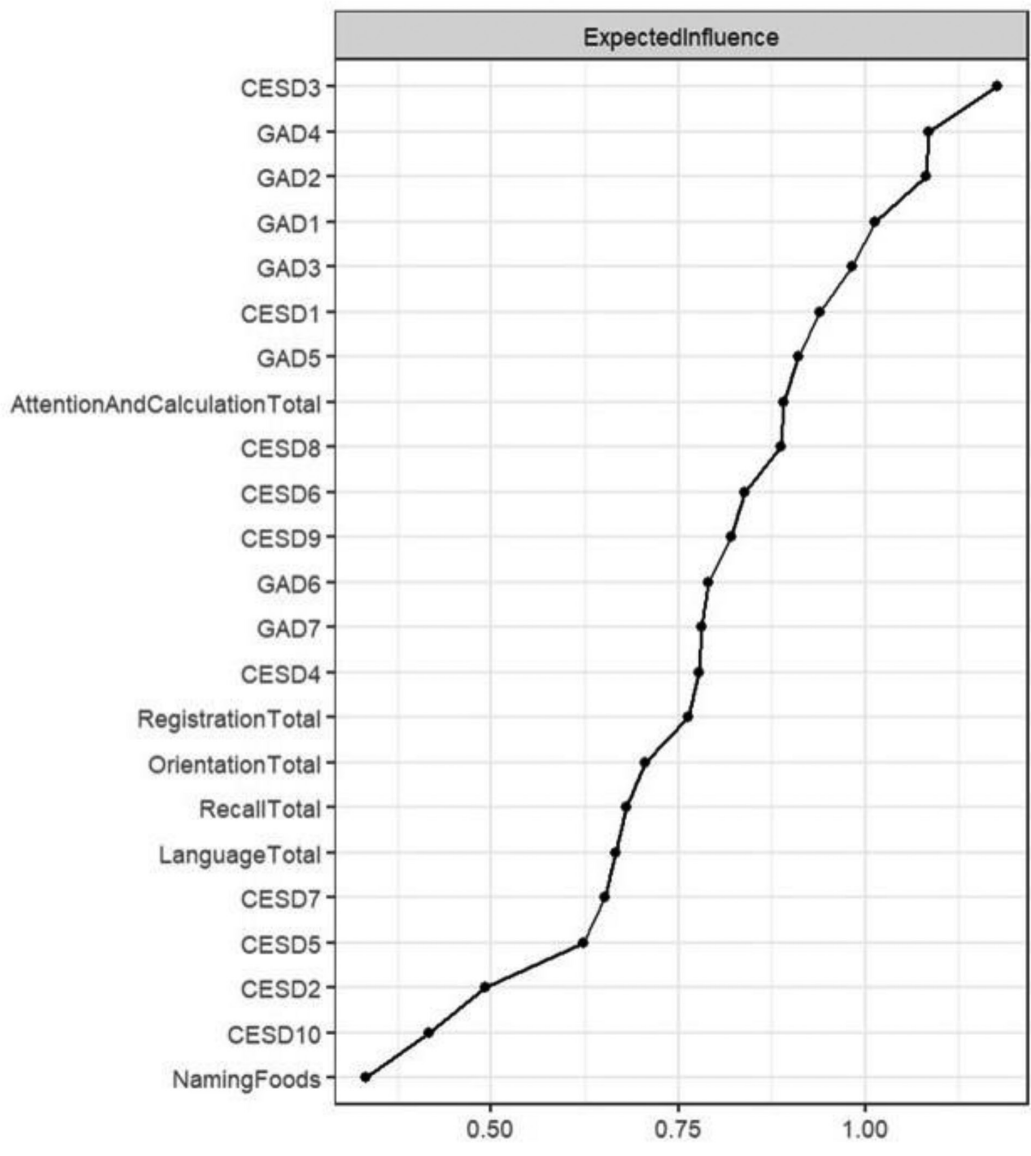
Network centrality of depressive and anxiety symptoms and cognition among the oldest-old population.

Figure 3 illustrates the top 3 central symptoms in the depressive, anxiety, and cognitive symptoms network structure, which are “Feeling blue/depressed” (CESD3), “Trouble relaxing” (GAD4) and “Uncontrollable worry” (GAD2). Results of the case-dropping bootstrap test show that the CS-coefficient of centrality Strength was 0.75, indicating that EI indices were reliable. The results of the nonparametric bootstrapped difference tests suggest that most of the node centrality and edge weights are significantly different from each other (Figs. S1, Supplemental Digital Content 1 and S3, Supplemental Digital Content 2). The bootstrapped 95% CIs of estimated edge weights are narrow, and most of the edge values are nonzero, meaning that most edges are reliable (Fig. S2, Supplemental Digital Content 3).

## 4. Discussion

The current study extends prior network analyses in Chinese elderly populations by integrating the prevalence and symptom-level network structure of depressive, anxiety, and cognitive symptoms specifically among Chinese adults aged 80 years and above. The focus on the oldest-old is clinically meaningful because this age group differs from younger-old adults in frailty, multimorbidity, bereavement, functional dependence, and survival selection, all of which may alter symptom profiles and intervention priorities. We found that the prevalence of depression was 67.4% (95% CI: 66.02%–68.69%) among adults aged over 80 years, which is significantly higher than the prevalence of 38.27% among older adults aged 60 years and above in China.^[9]^ Corresponding to our results is prior research based on data from the China Family Panel Studies, which reported that approximately 40% of older adults aged over 75 years exhibited significantly depressive symptoms.^[10]^ Considering the age differences of the samples in these studies, together these findings suggest that the prevalence of depression tends to increase with advancing age. It is possible that as individuals grow older, the likelihood of experiencing the loss of a spouse, close friends, or siblings increases, all of which contribute to prolonged periods of depressive symptoms.^[34]^ The identification of depressed mood as the most central symptom has practical or clinical implications. As is already known, psychiatric disorders are a result of interactions of various symptoms,^[35]^ depressed mood thus may play a critical role in eliciting emotional and cognitive problems among the oldest-old group. Clinically, therefore, interventions and treatments of depression could alleviate anxiety and enhance cognition for this group. Moreover, geriatric screening can also benefit from our finding in that many oldest-old citizens may not exhibit depressed symptoms when they are already victims of overall mental disease; therefore, screening programs are advised to prioritize depression even if it’s subthreshold. In 1 word, addressing depression will not only improve the emotional well-being of the oldest-old population but also preserve their cognitive functions.

Mild cognitive impairment, characterized as an intermediate and transitional stage between normal cognitive aging and dementia, is viewed as a critical issue among older adults.^[36]^ In our study, approximately 36.9% of participants were identified as having mild cognitive impairment (95% CI: 35.57–38.31%). In contrast, previous research reported a global prevalence of 15.56% (95% CI: 13.24–18.03%) among community-dwelling individuals aged 50 years and older.^[11]^ Age-related decline in brain function is particularly pronounced in regions such as the hippocampus, which is responsible for memory.^[37]^ The progressive loss of neurons and synaptic changes in these areas may contribute to the higher prevalence of cognitive impairment observed in the oldest-old population.^[38]^

In the depression–anxiety–cognition network model, the 3 psychological domains formed distinct clusters, but the strength of inter-cluster connections is too weak to be considered important. Though counterintuitive, such a finding is not without any support. In a longitudinal study on 8611 British people aged 50 and above, Gale et al revealed that most of the associations between depressive and cognitive symptoms only occurred among people aged below 80.^[15]^ Similarly, in another longitudinal study examining the relationships between depression, anxiety, psychological distress, and cognitive impairment among 352 Canadian older people aged 55 and above, Freire et al reported that anxiety was not able to predict cognitive decline, and that neither depression nor psychological stress alone was significantly associated with cognitive decline.^[39]^ A cross-sectional study covering 102 60–80-year-old American citizens also showed that without anxiety, depressive symptoms alone were not correlated with participants’ cognitive decline.^[40]^ A comparative analysis of these existing findings leads to a possible scenario, that is, depression, anxiety, and cognitive dysfunction may have plateaued out at some stage before 80, such that the interrelations are not strong enough to become significant.

Albeit the weak inter-cluster connections, some prominent nodes in the whole network structure are worth attention. The highest EI value falls on the node of “Feeling blue/depressed” (CESD3) in the network model, which demonstrates the core role of depression in the whole network model. Such a finding aligns with previous research conducted among diverse groups of older adults. For instance, Bai et al constructed a depression–cognition network model in older adults and found that “feeling blue/depressed” was the most central symptom.^[41]^ A systematic review of 17 network models examining the interrelations among symptoms of depression and anxiety identified “sad mood” as the most central symptom.^[42]^ According to the *Diagnostic and Statistical Manual of Mental Disorders, Fifth Edition* (DSM-5), “feeling depressed” is recognized as a core symptom for diagnosing major depressive disorder (American psychiatric association edition, 2013). In the oldest-old population, feelings of sadness or depression often arise from a complex interaction between physiological changes due to chronic illness and aging, along with psychosocial stressors common in later life. Targeting the “Feeling blue/depressed” may have practical significance in improving depression, anxiety and cognition for the oldest-old population.

Moreover, 2 nodes representing anxiety symptoms also play important roles in the depression–anxiety–cognition network, including “Trouble relaxing” (GAD4) and “Uncontrollable worry” (GAD2). “Uncontrollable worry” is defined as persistent concern over potential threats, which is linked to difficulty relaxing and represents a central emotional feature of anxiety disorders.^[43]^ Such findings corroborate earlier studies that identified “too much worry” and “uncontrollable worry” as core symptoms of generalized anxiety disorder.^[44,45]^ Among the oldest-old group, age-related functional declines are common. For example, sensory impairments, such as poor vision and hearing, can lead to misinterpretation, contributing to anxiety.^[46]^ Moreover, recent studies have shown that worry is linked to poor cognitive performance by consuming the mental resources needed for attention control.^[47,48]^ From a clinical perspective, interventions that address the central symptoms may help reduce the risk of subsequent depression and cognitive decline. Notably, multi-session cognitive bias modification has been shown to significantly alleviate anxiety and worry in patients with anxiety.^[49]^

The current study has several strengths, including a focus on the oldest-old population, a large sample size, and the application of a network approach to explore the core symptoms of depression, anxiety, and cognition. However, several limitations should also be acknowledged. First, due to the cross-sectional design, causal relationships between symptoms cannot be inferred; future cross-lagged network analyses based on longitudinal data are warranted. Second, the current study was conducted on the oldest-old population, which may limit the generalizability of the findings to other age groups or populations. Third, depressive and anxiety symptoms were assessed by self-report screening tools rather than diagnostic interviews, and measurement error may affect network estimation. Fourth, although the Chinese Longitudinal Healthy Longevity Survey has a national sampling design and includes residence-related variables, this symptom-level network did not explicitly model cultural or social-contextual factors such as filial piety, social support, widowhood, urban-rural resource differences, or healthcare access. Future studies should examine whether these factors moderate symptom networks and should use longitudinal or cross-lagged network models to clarify temporal pathways.

In summary, this study highlights “Feeling blue/depressed,” “Trouble relaxing,” and “Uncontrollable worry” as central symptoms within the depression–anxiety–cognition network. These symptoms should be considered key targets in the development of tailored interventions and preventive strategies for mental and cognitive health among the oldest-old population in China. Given the cross-sectional design, the possible mediating role of depressive symptoms between anxiety and cognitive decline should be tested in longitudinal data rather than inferred from the present network. Interventions such as cognitive behavioral therapy, particularly those targeting depressed mood through behavioral activation and cognitive restructuring, may be effective in alleviating both emotional and cognitive symptoms in this population.

## Author contributions

**Conceptualization:** Quan Zhou.

**Data curation:** Quan Zhou, Qianyu Zhao, Boning Zhang.

**Formal analysis:** Quan Zhou, Qianyu Zhao, Boning Zhang.

**Writing – original draft:** Quan Zhou, Qianyu Zhao, Boning Zhang.






